# High risk of short-term mortality and postoperative complications in patients with generalized peritonitis undergoing major emergency abdominal surgery—a cohort study

**DOI:** 10.1007/s00423-025-03637-4

**Published:** 2025-02-11

**Authors:** Maria Olausson, Mette A. Tolver, Ismail Gögenur

**Affiliations:** 1 Department of Surgery, Center for Surgical Science, Zealand University Hospital, Lykkebækvej 1, 4600 Køge, Denmark; 2 Department of Surgery, Zealand University Hospital, Køge, Denmark; 3 Department of Clinical Medicine, University of Copenhagen, Copenhagen, Denmark

**Keywords:** Secondary peritonitis, Perforation, Acute care surgery, Emergency surgery, Abdominal surgery, Laparotomy

## Abstract

**Background:**

Secondary generalized peritonitis is a potentially life-threatening condition. The aim of this study was to investigate the association between secondary generalized peritonitis and short-term mortality and postoperative complications in patients undergoing major abdominal emergency surgery.

**Methods:**

The study included patients with the age ≥ 18 years undergoing major emergency abdominal surgery in a University Hospital from 2017 to 2019 after the introduction of a perioperative bundle care program. The primary outcome measures were short-term mortality, defined as death within 30 and 90 days after surgery and postoperative complications within 30 days after surgery. Uni- and multivariable logistic regression analyses were performed to evaluate risk factors for 30- and 90-days mortality and 30-days postoperative complications.

**Results:**

A total of 591 patients were included, of whom 21% (124/591) had generalized peritonitis. The overall 30 day-mortality rate was 12.5% (74/591). Patients with generalized peritonitis had a significantly higher 30-day mortality rate than patients with non-generalized peritonitis 18.5% (23/124) vs. 10.9% (51/467), *P* = 0.033. Generalized peritonitis was an independent risk factor for 30- and 90- days mortality. There was a significantly higher rate of admission to ICU for patients with generalized peritonitis 39.5% (49/124) vs. 12.6% (59/467), *P* < 0.001. Patients with generalized peritonitis had significantly higher rates of surgical and non-surgical complication compared to patients with non-generalized peritonitis 87.1% (108/124) vs. 65.7% (307/467), *P* < 0.001. Generalized peritonitis was an independent risk factor of 30 days postoperative complications.

**Conclusion:**

In a population undergoing major emergency abdominal surgery treated in a perioperative optimization protocol, generalized peritonitis was an independent risk factor for both 30- and 90-days mortality and postoperative complications.

## Introduction

Secondary peritonitis is the second leading cause of sepsis and generalized peritonitis has reported mortality rates as high as 20% with an increasing risk of mortality and complications depending on the severity [1–4]. Secondary peritonitis is characterized as inflammation of the abdominal lining caused by a disruption of the gastrointestinal tract or in some cases bowel ischemia or obstruction. Secondary peritonitis can be present in a mild form either as serous liquid or localized contamination as non-generalize peritonitis or in worst cases involve the entire abdominal cavity generating generalized peritonitis [3, 5]. The severity of the condition depends on factors such as the disease pathology, extent of contamination, timing of interventions such as source control and antibiotics, and patient related factors [6–9]. The inflammatory response generated by secondary peritonitis can lead to severe systemic inflammation, sepsis, and multiorgan dysfunction, making early recognition and aggressive management critical [6, 10–12].

The population of patients undergoing major emergency abdominal surgery is complex due to heterogeneity and often seen with multiple comorbidities, making the risk profile multifaceted. Therefore, identifying key predictors of poor outcome and developing preoperative scoring systems for mortality and morbidity are essential to guide the implementation of multidisciplinary perioperative care bundles, ultimately optimizing the perioperative pathway for this patient population. Recent research has demonstrated the predictive significance of peritoneal contamination, showing that the extent and nature of contamination influence outcomes [13]. Prediction tool such as Mannheim Peritonitis Index (MPI), World Society of Emergency Surgery (WSES) sepsis severity score, National Emergency Laparotomy Audit (NELA) risk calculator, Physiological and Operative Severity Score for the enUmeration of Mortality and Morbidity (POSSUM), integrate both contamination severity and patient related factors, to risk stratify and predict mortality outcome [14]. Despite many of these risk assessment tools performing well, they are often comprehensive, making their applicability in the daily practice in the clinic challenging. Nonetheless, the prediction tools and literature on secondary peritonitis support the importance of recognizing peritonitis in predicting and addressing surgical risks to guide clinical decisions and improve perioperative management. Hence, there is still a need to identify the patients with the need of a more tailored treatment course and to identify whether new treatment approaches can modulate outcomes through e.g. alternative routes of antibiotic administration and stress modification through perioperative treatment with inflammatory modulators. Considering the increased inflammation and systemic impact of generalized peritonitis, we hypothesized that patients with generalized peritonitis undergoing surgery in an enhanced perioperative multimodal protocol has the highest risk of mortality and complications in the first 90 days after surgery as opposed to patients with non-generalized peritonitis. We therefore aimed to report short term outcomes in patients undergoing surgery for generalized secondary peritonitis, and to investigate independent risk factors for both outcomes in patients undergoing emergency abdominal surgery in an enhanced perioperative multimodal protocol.

## Mterials and methods

### Study design and setting

The study was a retrospective observational cohort study using prospectively collected data during implementation of a local high quality multidisciplinary standardized protocol for major emergency abdominal surgical patients at the Department of Surgery, Zealand University Hospital, in a two-year period from March 2017 to February 2019 [15]. The study was approved by the Head of the Department of Surgery and by the Danish Data Protection agency (no. REG-080–2022) and did not qualify for ethics approval by Danish law. The study followed the standards of reporting of observational studies in epidemiology (STROBE) statements [16].

### Study population

The study included patients with the age ≥ 18 years undergoing major emergency abdominal surgery. Major emergency abdominal surgery was performed within 72 h of admission to the Department of Surgery. Patents undergoing surgery for obstruction, perforation, ischaemia, non-traumatic intraabdominal bleeding or abscess, or other diagnosis requiring emergency laparoscopy or laparotomy were included. Additionally, patients with malignancy requiring palliative laparoscopy or laparotomy were also included. Patients involved in trauma, primary laparoscopic appendectomy and cholecystectomy, hernia without bowel obstruction, or any elective surgery were not included in the study. However, if reoperated due to complication from a non-emergency surgery and meeting the above criteria they were included.

All patients received the same high quality standardized protocol with a care bundle covering surgical, emergency, anaesthesiologic, radiological, physiotherapy, and nutritional support. It contains items of pre-, intra-, and postoperative interventions including rapid diagnosis, resuscitation, surgical treatment, and optimizing care postoperatively (Box 1). All patients were handled by attending or consultant physicians in all the departments. A comprehensive item list is described in detail elsewhere [15].

**Box 1**

**Table:** 

Care bundle items in optimizing major emergency abdominal surgery
Preoperative initiatives: If clinical suspicion of the need for major emergency abdominal surgery • Prophylactic AB and fluid resuscitation at admission • CT scan within 2 h • Surgery within 6 h
Intraoperative initiatives: • Surgery performed with attending or consultant present during entire surgical procedure • Perioperative thoracic epidural • Postoperative risk scoring
Postoperative initiatives: • Standardized PACU discharge to surgical ward • Standard postoperative biochemistry • Standard postoperative medication • Postoperative physiotherapeutic assessment • Postoperative nutritional screening and intervention admitted through the emergency department directly to the department of surgery

*AB* antibiotics, *CT* computer tomography, *PACU* post-anaesthesia care unit

Patients were categorized into two groups according to the perioperative peritonitis status assessed during the surgical procedure. The non-generalized group included no peritonitis findings, serous peritonitis, or local contamination, and the generalized peritonitis included findings of blood, purulent, or faecal contamination of ≥ 2 intraabdominal quadrants.

All patients from March 2017 who met the above-mentioned criteria received the same standardized pre, intra, and post-operative protocol (Box 1). The patients included in the study had no missing data on peritonitis status, mortality, or postoperative complication assessments.

### Outcomes

The primary outcome measures were short-term mortality, defined as death within 30 and 90 days after surgery and postoperative complications within 30 days after surgery. All patients were followed up until one year after surgery to evaluate vital status. Death of all citizens is registered in the Danish Civil Registration System with a 100% follow-up and is automatically linked with the electronic patient record. Thirty days postoperative complications were evaluated by the investigators and classified according to the Clavien-Dindo Classification system and defined as Clavien-Dindo score ≥ 3b [17].

Secondary outcomes measures were rates of in-hospital mortality, 180 days mortality, length of stay (LOS), readmission within six month of discharge, intensive care unit (ICU) admission and length of ICU stay, reoperation, and postoperative complications. Additional outcomes were risk factors associated with the short-term mortality and postoperative complications.

### Data collection

All data were obtained from the electronic patient records prospectively and stored in an electronic case report form. The following demographics and preoperative variables included: sex, age, body mass index (BMI) kg/m^2^, smoking status, alcohol consumption above recommendation (84 g/week for women and 168 g/week for men), non-steroidal anti-inflammatory drugs (NSAID) and glucocorticoids (systemic) usage, American Association of Anaesthesiologists classification (ASA) [18], WHO performance score [19], and comorbidities. Investigators calculated Charlson Comorbidity Index (CCI) [20] and quick Sequential Organ Failure Assessment (qSOFA) [21]. Intraoperative variables included: length of surgical procedure, operative findings (obstruction, perforation, intraabdominal bleeding, ischemia or necrosis, anastomotic leakage, other findings, and no pathology), perioperative malignancy (yes or no), type of surgery (laparoscopy, laparoscopy converted to open, and laparotomy), surgical procedure (procedures without resection, upper GI procedure, small bowel procedure, small- and bowel procedure, large bowel and rectal procedure, and other procedure), reoperation after non-emergency surgery (yes or no), blood transfusion (yes or no).

### Statistics

Categorical data were presented as number of cases and frequency in percentages and group comparisons were analysed with a chi-squared test or a Fisher’s exact test. The data were presented as mean with standard deviation or median and interquartile range (IQR) for numerical data. Variables, age, BMI, length of surgical procedure, length of ICU stay, and length of hospital stay, were analysed with unpaired t-test or Mann–Whitney’s test according to data distribution for group comparisons. The association between pre- and intraoperative variables and the risk of 30- and 90-days mortality and 30-days postoperative complications were analysed with uni- and multivariable logistic regression analyses. Variables chosen for the logistic regression were based on prior research and included sex, age > 70 years, active smoking, ASA ≥ 3, WHO PS ≥ 2, CCI ≥ 3, laparotomy, generalized peritonitis, perioperative malignancy, and reoperation after non-emergency surgery. All included variables were tested for multicollinearity by assessing correlation and variance inflation factor (VIF). From the univariable analysis the variables with *P*-values < 0.2 were included in the multivariable analysis. Results were expressed as unadjusted and adjusted odds ratios (OR) with a 95% confidence interval (95% CI). The adjusted odds ratios are presented in Forest plots.

*P*-value < 0.05 was considered statistically significant. All statistics were performed in R Studio version 4.2.2 (R Foundation for Statistical Computing, Vienna, Austria).

## Results

**Fig. 1:**
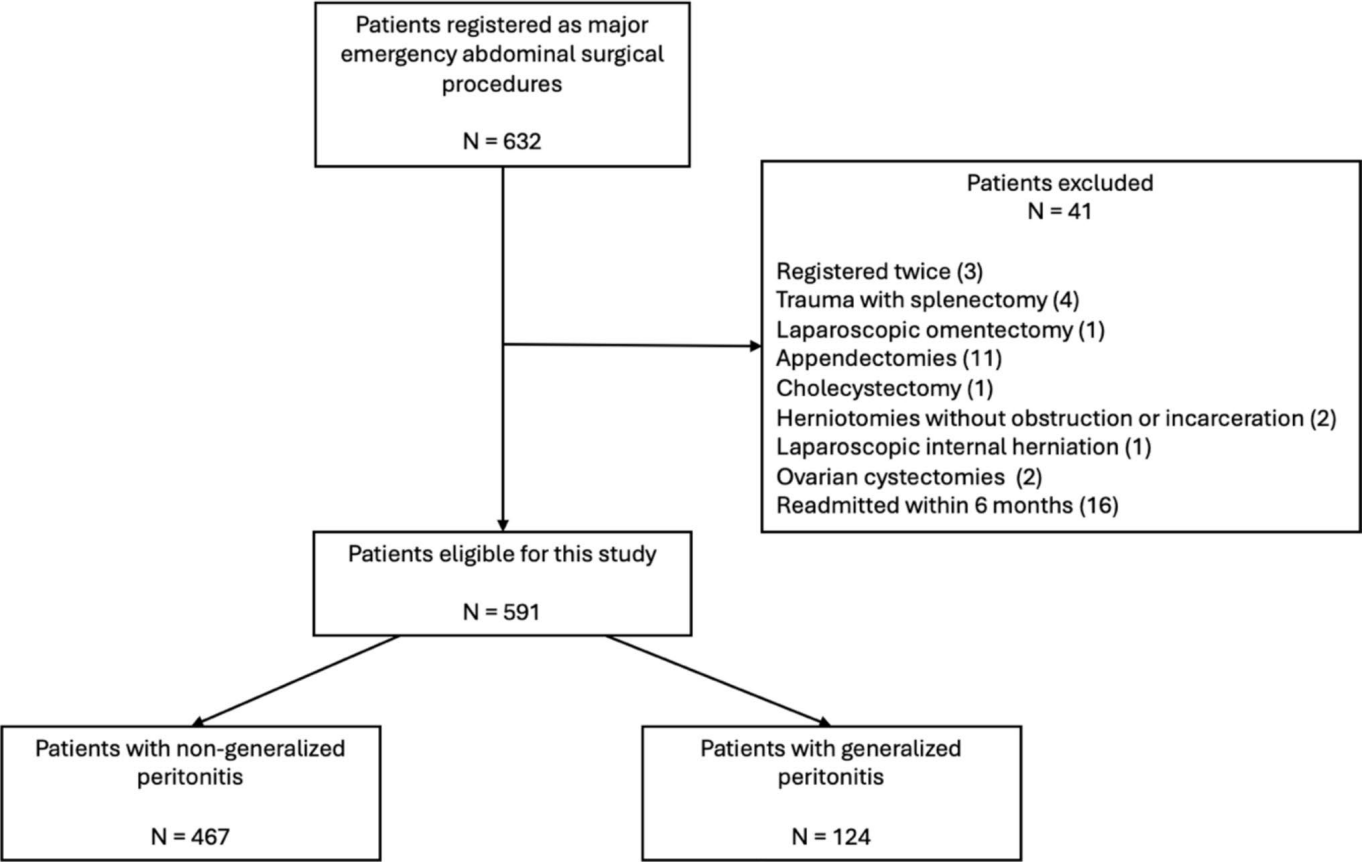
Flowchart

In a two-year study period, 632 eligible patients underwent major emergency abdominal surgery. Forty-one patients were not included in the current study, as patients did not meet the diagnosis and surgical procedure criteria of major emergency abdominal surgery protocol, leaving a total of 591 included patients (Fig. 1).

Patient demographics are presented in Table 1. A total of 21% (124/591) had an intraoperative finding of generalized peritonitis. Patients with generalized peritonitis were more frequently active smokers, had an alcohol consumption above recommendation, and had a q-SOFA score ≥ 1 at admission compared to patients without generalized peritonitis (Table 1).

**Table 1:** Demographics and clinical characteristics of patients undergoing major emergency abdominal surgery

	Non-generalized peritonitis *n* = 467	Generalized peritonitis *n* = 124	*P* value
Male sex, *n* (%)	252 (52.6)	53 (40.8)	0.058
Age, median [IQR]	69 [57, 78]	71 [61, 78]	0.179
BMI, median [IQR]	24.60 [22.00, 29.20]	24.75 [21.42, 28.22]	0.480
Active smoking, *n* (%)	102 (22.4)	45 (37.2)	**0.001**
Alcohol above recommendation, *n* (%)	40 (8.7)	24 (19.7)	**0.001**
ASA, *n* (%)			0.809
1	67 (14.3)	15 (12.1)	
2	197 (42.2)	50 (40.3)	
3	175 (37.5)	52 (41.9)	
≥ 4	28 (6.0)	7 (5.6)	
WHO PS, *n* (%)			0.186
0	230 (49.3)	52 (41.9)	
1	140 (30.0)	41 (33.1)	
2	58 (12.7)	24 (19.4)	
3	28 (6.0)	6 (4.8)	
≥ 4	11 (2.4)	1 (0.8)	
Charlson Comorbidity Index, *n* (%)			0.235
0	49 (10.5)	13 (10.5)	
1	54 (11.6)	8 (6.5)	
2	68 (14.6)	14 (11.3)	
≥ 3	296 (63.4)	89 (71.8)	
Cardiovascular diseases, *n* (%)	228 (48.8)	64 (51.6)	0.652
Diabetes mellitus, *n* (%)	57 (12.2)	15 (12.1)	1.000
Pulmonary diseases, *n* (%)	70 (15.0)	17 (13.7)	0.830
Chronic kidney disease, *n* (%)	30 (6.4)	6 (4.8)	0.656
Gastrointestinal diseases, *n* (%)	53 (11.3)	9 (7.3)	0.247
Malignancy, *n* (%)			0.089
None	325 (69.6)	87 (70.2)	
Active	67 (14.3)	25 (20.2)	
Previous	75 (16.1)	12 (11.7)	
Glucocorticoids, *n* (%)	30 (6.4)	7 (5.6)	0.913
NSAIDs, *n* (%)	38 (7.9)	14 (11.3)	0.314
qSOFA, *n* (%)			**0.006**
0	349 (83.9)	76 (69.7)	
1	61 (14.7)	28 (25.6)	
2	4 (1.0)	3 (2.8)	
3	2 (0.5)	2 (1.8)	

*BMI* body mass index, *ASA* American society of anesthesiologists, *PS* performance status, *NSAID* non-steroidal anti-inflammatory drugs, *qSOFA* quick sequential organ failure assessment score

Patients with generalized peritonitis were significantly more likely to have operative findings such as perforation 74.2% (92/124) vs. 10.3% (48/467) *P* < 0.001 and intraabdominal bleeding 4.8% (6/124) vs. 1.1% (5/467) *P* = 0.017, compared to the non-peritonitis group (Table 2). There was no significant difference in perioperative malignancy between the groups (*P* = 1.000).

**Table 2:** Perioperative findings of patients undergoing major emergency abdominal surgery

	Non-generalized peritonitis *n* = 467	Generalized peritonitis *n* = 124	*P* value
Duration of surgery, median [IQR]	139.00 [95.00, 194.50]	150.00 [110.00, 204.00]	0.111
Operative findings, *n* (%)			
Obstruction	343 (73.4)	13 (10.5)	** < 0.001**
Perforation	48 (10.3)	92 (74.2)	** < 0.001**
Intraabdominal bleeding	5 (1.1)	6 (4.8)	**0.017**
Leakage	6 (1.3)	3 (2.4)	0.614
Bowel ischemia	24 (5.1)	8 (6.5)	0.726
Other	30 (6.4)	2 (1.6)	0.060
No pathology	11 (2.4)	0 (0.0)	0.177
Perioperative malignancy, *n* (%)	52 (11.1)	14 (11.3)	1.000
Type of surgery, *n* (%)			
Laparoscopy	48 (10.3)	19 (15.3)	0.157
Laparoscopy converted to open	66 (14.1)	40 (32.3)	** < 0.001**
Laparotomy	353 (75.6)	65 (52.4)	** < 0.001**
Surgical procedure, *n* (%)			
Procedures without resection	281 (60.2)	22 (17.7)	** < 0.001**
Upper GI	17 (3.6)	34 (27.4)	** < 0.001**
Small bowel resection	77 (16.5)	20 (16.1)	1.000
Small and large bowel resection	40 (8.6)	11 (8.9)	1.000
Large bowel resection	39 (8.4)	36 (29.0)	** < 0.001**
Other procedures	13 (2.8)	1 (0.8)	0.340
Reoperation after non-emergency surgery, *n* (%)	26 (5.6)	14 (11.3)	**0.041**
Perioperative transfusion, *n* (%)	51 (10.9)	29 (23.4)	**0.001**

*GI* gastrointestinal

Patients with generalized peritonitis had a significantly higher rate of conversion to open approach 32.3% (40/124) compared to patients with non-generalized peritonitis 14.1% (66/467) *P* < 0.001. However, there was no difference in the length of surgery in the two groups (*P* = 0.111). Furthermore, there was a significant difference in the specific surgical procedure between the generalized peritonitis group and non-generalized peritonitis group (*P* < 0.001). Patients with generalized peritonitis were significantly more likely to have a procedure performed on the upper GI tract 27.4% (34/124) vs. 3.6% (17/467) *P* < 0.001, or large bowel resection 29% (36/124) vs. 8.4% (39/467) *P* < 0.001. Patients with non-generalized peritonitis had a significant higher rate of procedures not requiring bowel resection 60.2% (281/467) vs. 17.7% (22/124) *P* < 0.001. When looking at surgery due to complication from a primary non-emergency procedure, this was more common in the generalized peritonitis group 11.3% (14/124) vs. 5.6% (26/467) *P* = 0.041. Moreover, blood transfusion perioperatively were likewise significantly more common in the generalized peritonitis group 23.4% (29/124) vs. 10.9% (51/467) *P* = 0.001 (Table 2).

Looking postoperatively, patients with generalized peritonitis had a significant longer length of hospital stay with a median of 9.7 days versus 6.5 days (*P* < 0.001). There were no significant differences on readmissions within 6 months in patients with and without generalized peritonitis (45.5% vs. 47.9%, *P* = 0.707). There was a significantly higher rate of admission to ICU for patients with generalized peritonitis 39.5% (49/124) vs. 12.6% (59/467), *P* < 0.001, but there were no differences in the length of ICU admission (*P* = 0.202). When looking at postoperative complications of any kind, patients with generalized peritonitis had significantly higher occurrence of both surgical and non-surgical complications compared to patients with non-generalized peritonitis (Table 3). The overall 30 day-mortality rate was 12.5% (74/591). Patients with generalized peritonitis had a significantly higher mortality rate in both overall mortality, in-hospital mortality, 30-days mortality, 90-days mortality, and 180-days mortality (Table 3).

**Table 3:** Postoperative outcomes of patients undergoing major emergency abdominal surgery

	Non-generalized peritonitis *n* = 467	Generalized peritonitis *n* = 124	*P* value
Length of admission, median [IQR]	6.49 [3.89, 11.79]	9.70 [5.16, 15.69]	** < 0.001**
Readmission after discharge, *n* (%)	222 (47.9)	56 (45.5)	0.707
Admission to ICU, *n* (%)	59 (12.6)	49 (39.5)	** < 0.001**
Length of ICU admission, median [IQR]	3.44 [1.46, 5.79]	3.39 [1.99, 8.35]	0.202
Any postoperative complications, n (%)	307 (65.7)	108 (87.1)	** < 0.001**
Clavien Dindo Classification, *n* (%)			**0.003**
CD 1	13 (4.2)	3 (2.8)	
CD 2	77 (25.1)	19 (17.6)	
CD 3a	85 (27.7)	20 (18.5)	
CD 3b	48 (15.6)	16 (14.8)	
CD 4a	23 (7.5)	14 (13.0)	
CD 4b	10 (3.3)	13 (12.0)	
CD 5	51 (16.6)	23 (21.3)	
Reoperation, *n* (%)	112 (24.0)	48 (38.7)	**0.002**
Surgical complications, *n* (%)	119 (25.5)	56 (45.2)	** < 0.001**
Cardiovascular complications, *n* (%)	110 (23.6)	53 (42.7)	** < 0.001**
Pulmonary complications, *n* (%)	137 (29.3)	57 (46.0)	**0.001**
Acute kidney injury, *n* (%)	9 (1.9)	8 (6.5)	**0.017**
Mortality *n* (%)			
In hospital	39 (8.4)	23 (18.5)	**0.002**
30-days mortality	51 (10.9)	23 (18.5)	**0.033**
90-days mortality	74 (15.8)	34 (27.4)	**0.005**
180-days mortality	85 (18.2)	39 (31.5)	**0.002**

*ICU* intensive care unit

**Fig. 2:**
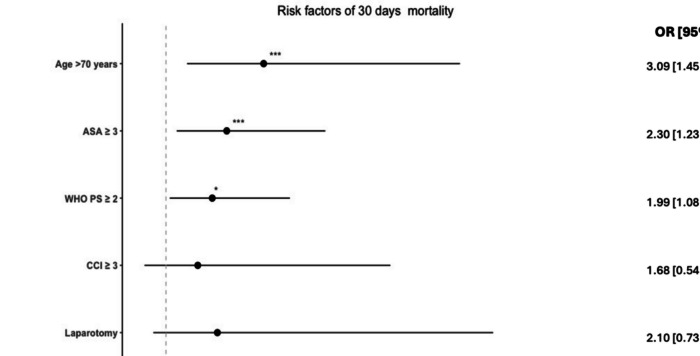
Forest plot of multivariable logistic regression analysis of risk factors associated with 30 days mortality

Univariable logistic regression analysis showed that, age > 70, ASA ≥ 3, WHO PS ≥ 2, CCI ≥ 3, laparotomy, generalized peritonitis, and perioperative malignancy were significantly associated with the risk of 30-days mortality. In the multivariable analysis, age > 70, ASA ≥ 3, WHO PS ≥ 2, generalized peritonitis, and perioperative malignancy were independently associated with risk of 30-days mortality (Fig. 2).

**Fig. 3:**
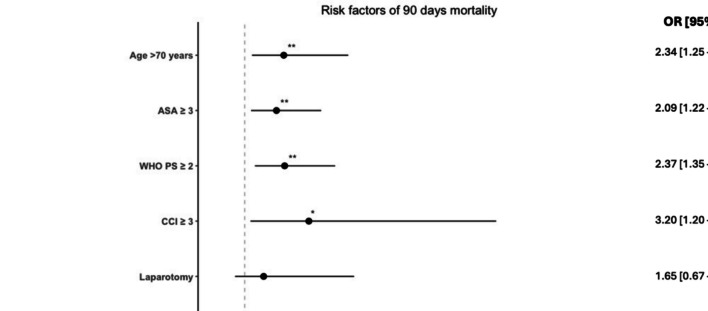
Forest plot of multivariable logistic regression analysis of risk factors associated with 90 days mortality

The logistic regression analysis for 90 days-mortality including the same covariates as above and re-operation after non-emergency surgery showed that, age > 70, ASA ≥ 3, WHO PS ≥ 2, CCI ≥ 3, generalized peritonitis, and perioperative malignancy were significantly associated with the risk of 90-days mortality. Similar to 30-days mortality, in the multivariable analysis 90-days mortality showed the same independent risk pattern however with CCI ≥ 3 as an additional risk factor (Fig. 3).

**Fig. 4:**
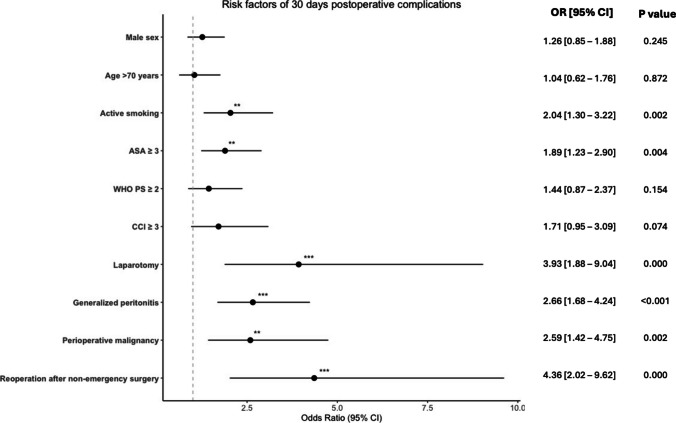
Forest plot of multivariable logistic regression analysis of risk factors associated with 30 days postoperative complications, Clavien Dindo ≥ 3b

Univariable logistic regression on 30-days postoperative complications Clavien-Dindo ≥ 3b showed that male sex, age > 70, active smoking, ASA ≥ 3, WHO PS ≥ 2, CCI ≥ 3, laparotomy, generalized peritonitis, perioperative malignancy, and reoperation after non-emergency surgery were significantly associated with 30-days postoperative complications Clavien Dindo ≥ 3. In the multivariable analysis, active smoking, ASA ≥ 3, laparotomy, generalized peritonitis, perioperative malignancy, and reoperation after non-emergency surgery were independently associated with risk of postoperative complications (Fig. 4).

## Discussion

In this retrospective cohort study, we investigated 591 patients undergoing major emergency abdominal surgery in a well-established perioperative multidisciplinary protocol based on Enhanced Recovery After Surgery (ERAS) principles [22–24]. One fifth of the patients had generalized peritonitis, and they were significantly more likely to get admitted to ICU, undergo a surgical reintervention, have a longer admission, and in higher frequency of both postoperative medical and surgical complications. About one fifth of the patients with generalized peritonitis died within 30 days, which was almost twice as many as the patients with non-generalized peritonitis. Furthermore, other common risk factors for complications and mortality were age > 70, generalized peritonitis, and perioperative malignancy.

In our study, the overall 30-days mortality rate was 12.5%. Similar perioperative settings in Denmark have shown mortality rates ranging from 6.7–15.5% [25–28]. Outside Denmark 30-days mortality rates have been reported between 10.5–14.9% [29–31]. The variation in mortality rates is multi-faceted but factors such as population heterogenicity, disease pathology, and patient-related factors are important contributors [3, 5]. Furthermore, the organisational and clinical resources within the emergency setting also seem to have impact on mortality and negative postoperative outcome [32, 33].

Procedures like cholecystectomies, appendectomies, and traumas were not included in the current study, which potentially contributes to a higher overall mortality rate within the population compared to other studies. Moreover, reporting of an overall 30-days mortality rate for patients undergoing major emergency abdominal surgery may not show the whole picture of this population. By categorizing patients into generalized peritonitis and non-generalized peritonitis, the mortality rate for patients with generalized peritonitis was significantly higher at both 30-, 90-, and 180-days (Table 3). Mortality rates in other studies investigating secondary peritonitis have found overall mortality ranging from 17.5–30% [6, 34, 35].

Generalized peritonitis was an independent risk factor for both 30- and 90-days mortality and 30-days postoperative complications in multivariable analyses suggesting that these patients may represent a subpopulation that should be targeted for optimization of innovations in perioperative care. Similar results have been reported in several studies looking at pre- and intra-operative factors influencing for mortality. A prospective multicentre study in France including 14 departments concluded that generalized peritonitis was an independent risk factor for both 28-day mortality and length of stay [36]. A prospective multinational study conducted in ICU departments showed that aside from age > 60 years, diffuse peritonitis was among the independent risk factors for 28-day mortality [37]. In a recent multicentre study from Senegal, generalized peritonitis was also an independent predictor for 30-day mortality [38].

The population of emergency abdominal surgery is heterogenic; but generalized peritonitis is an important contributor when investigating prognostic variables in short-term mortality worldwide. Preoperative scoring systems such as Boey score [39], PULP score [40], and Jabalpur score [41] have demonstrated an area under the curve (AUC) above 80% in predicting mortality in patients with perforated peptic ulcers (PPU) [42]. However, when applied to patients with general peritonitis without specified aetiology, especially Jabalpur score did not perform well with an AUC below 0.60 [43]. One study has tried to establish an intraperitoneal contamination index with only biomarker reaching an AUC of 0.73 and 0.78 for purulent and faecal contamination, however this was only in patients undergoing laparotomy and the extension of contamination was not assessed [44]. In order to incorporate the aetiology of peritoneal contamination, intraoperative factors must be assessed. To our knowledge the only risk assessment tool looking more specifically into the origin and extension of secondary peritonitis combined with preoperative factors is the MPI score, however it has shown large variation in the ability to accurately predict mortality with an AUC varying from 0.69–0.95 [14, 43, 45–47]. Other well-known risk assessment tools such as POSSUM, NELA, and WSES, are applicable for a broader surgical population, however they are more complex requiring information not always available or tedious to acquire. Choosing the right scoring system, risk calculator or model can be extremely difficult and one size fits all may not apply to a population undergoing major emergency adnominal surgery with such a huge heterogeneity. Prediction models only taking preoperative factors into account will lack important knowledge about the extension and source of peritoneal contamination, however, may be good enough when used for specific patient populations. On the other hand, incorporating too many factors over the course of the perioperative period may reduce the applicability for the general clinician, but seems to aim for a broader patient population and can be more goal-directed.

Apart from generalized peritonitis, age above 70 years, ASA ≥ 3, WHO PS ≥ 2, CCI ≥ 3 and perioperative malignancy were strongly associated with mortality. A study from the NELA population also reported age, ASA and perioperative malignancy being a predictor of 30 days mortality [48]. Another study reported similar results, where ASA was a predictor for in-hospital and 30-day mortality, and malignancy was found predictive for one year mortality [49]. This is also seen in a Danish study including 4336 patients undergoing emergency abdominal surgery [28]. Other studies found age down to 60 years was an independent risk factor of 30-days mortality [28, 37]. WHO PS ≥ 2 was associated with mortality, which correspond to findings of previous studies evaluating outcomes after emergency surgery, some including not only performance status, but also frailty scores [50–52]. Not surprisingly, it seems that these factors are strong independent risk factors of short-term mortality followed by the presence of generalized peritonitis. Additionally, we found active smoking, ASA ≥ 3, laparotomy, and reoperation after non-emergency surgery as strongly associated with 30-days postoperative complications.

The window for optimization is short in emergency surgery and the cornerstone in treatment of secondary peritonitis is rapid antibiotics, resuscitation, and surgical source control [3, 23]. Little has changed in this modality over the years, although minimal invasive surgery, pain management, and the use of steroids have had some effects in reducing the surgical stress response and thereby mortality and morbidity [53–55]. However, this study indicates the need to rethink possible new interventions, to reduce mortality and morbidity in patients with generalized secondary peritonitis. We have identified four risk factors which both accounts for short term mortality and postoperative complications; Age above 70 years, ASA score above 2, generalized peritonitis, and perioperative malignancy which we cannot alter prior to surgery. However, generalized peritonitis has the potential to be modulated by alternative routes of antibiotic administration, and stress modification through perioperative treatment with inflammatory modulators. A study done on complicated appendicitis investigated treatment with granulocyte–macrophage colony-stimulating factor (GM CSF) administrated intraperitoneally and showed promising results in reducing length of stay and postoperative complications [56]. Further, a recent randomized control trial found that a single preoperative high dose of dexamethasone significantly reduced the inflammatory response after major emergency surgery including patients with peritonitis [57]. Potential future studies are needed to investigate how to reduce the inflammatory response of generalized peritonitis using the intraoperative window.

This study represents real-world conditions and practices, which enhance the external validity making them more applicable to the general population. On the contrary, the study is a single-centre retrospective study done in a highly optimized protocol for emergency abdominal surgery limiting generalizability. There is a potential for selection bias, as the cohort is determined by those who underwent surgery and may not represent all emergency abdominal cases, however all patients undergoing surgery received the same treatment and only few was excluded after surgery as they did not meet the inclusion criteria. Additionally, allocation bias may be present as peritonitis status is based on subjective evaluation of patient reports, nevertheless all patient reports were assessed by the same investigator. Furthermore, we have tested the confounding variables for collinearity with correlation and variance inflation factor analyses, showing acceptable values. Adjustments for multiple testing were not made due to the rational that some of the variables are repeated in the different analyses and many of the variables are interrelated and the findings are shown in prior research providing association. Doing adjustment for multiple testing might be too stringent and could increase type 2 errors and cover possible known associations. Lastly, instead of age as a prognostic factor, a more modern variable such as frailty score would have been preferred, as it is more related to age-related physiological decline rather than age alone.

## Conclusions

In conclusion, this study found that generalized peritonitis was an independent risk factor for both 30- and 90-days mortality and postoperative complications within a population undergoing major emergency abdominal surgery. Additional factors associated with mortality were age, ASA score, WHO PS, CCI, and perioperative malignancy. Moreover, the study showed the same risk profile between 30- and 90-days mortality, though generalized peritonitis was more strongly associated with 90-days mortality and even more with 30-days postoperative complications, highlighting that the extension of peritoneal contamination is a valuable factor in the patient’s postoperative trajectory. Patients with generalized peritonitis may be in need of further optimization potentially gaining effect of an add-on treatment regime.

## Data Availability

According to Danish law about data protection the data material cannot be shared.
